# Genetic Variants Associated with Increased Risk of Malignant Pleural Mesothelioma: A Genome-Wide Association Study

**DOI:** 10.1371/journal.pone.0061253

**Published:** 2013-04-23

**Authors:** Giuseppe Matullo, Simonetta Guarrera, Marta Betti, Giovanni Fiorito, Daniela Ferrante, Floriana Voglino, Gemma Cadby, Cornelia Di Gaetano, Fabio Rosa, Alessia Russo, Ari Hirvonen, Elisabetta Casalone, Sara Tunesi, Marina Padoan, Mara Giordano, Anna Aspesi, Caterina Casadio, Francesco Ardissone, Enrico Ruffini, Pier Giacomo Betta, Roberta Libener, Roberto Guaschino, Ezio Piccolini, Monica Neri, Arthur W. B. Musk, Nicholas H. de Klerk, Jennie Hui, John Beilby, Alan L. James, Jenette Creaney, Bruce W. Robinson, Sutapa Mukherjee, Lyle J. Palmer, Dario Mirabelli, Donatella Ugolini, Stefano Bonassi, Corrado Magnani, Irma Dianzani

**Affiliations:** 1 Human Genetics Foundation, HuGeF, Turin, Italy; 2 Department of Medical Sciences, University of Turin, Turin, Italy; 3 Laboratory of Genetic Pathology, Department Health Sciences, University of Piemonte Orientale, Novara, Italy; 4 CPO-Piemonte and Unit of Medical Statistics and Epidemiology, Department Translational Medicine, University of Piemonte Orientale, Novara, Italy; 5 Genetic Epidemiology and Biostatistics Platform, Ontario Institute for Cancer Research, Toronto, Ontario, Canada; 6 Prosserman Centre for Health Research, Samuel Lunenfeld Research Institute, Toronto, Ontario, Canada; 7 Centre for Genetic Epidemiology and Biostatistics, University of Western Australia, Nedlands, Western Australia, Australia; 8 Centre of Expertise for Health and Work Ability, Finnish Institute of Occupational Health, Helsinki, Finland; 9 Laboratory of Genetics, Department Health Sciences, University of Piemonte Orientale, Novara, Italy; 10 Thoracic Surgery Unit, University of Piemonte Orientale, Novara, Italy; 11 Chest Surgery, Department of Clinical and Biological Sciences, University of Turin, Orbassano, Italy; 12 Thoracic Surgery Unit, University of Turin, Turin, Italy; 13 Pathology Unit, Azienda Ospedaliera Nazionale SS, Antonio e Biagio e Cesare Arrigo, Alessandria, Italy; 14 Transfusion Centre, Azienda Ospedaliera Nazionale SS, Antonio e Biagio e Cesare Arrigo, Alessandria, Italy; 15 Pneumology Unit, Santo Spirito Hospital, Casale Monferrato, Italy; 16 Unit of Clinical and Molecular Epidemiology IRCCS San Raffaele Pisana, Rome, Italy; 17 Department of Respiratory Medicine, Sir Charles Gairdner Hospital, Nedlands, Western Australia, Australia; 18 National Centre for Asbestos Related Disease, School of Medicine and Pharmacology, University of Western Australia, Nedlands, Western Australia, Australia; 19 Centre for Child Health Research, The University of Western Australia, Nedlands, Western Australia, Australia; 20 PathWest Laboratory Medicine WA, Nedlands, Western Australia, Australia; 21 Department of Medicine, University of Toronto, Toronto, Ontario, Canada; 22 Women's College Research Institute and Women's College Hospital, Toronto, Ontario, Canada; 23 Unit of Cancer Epidemiology, CPO-Piemonte and University of Turin, Turin, Italy; 24 Interdepartmental Center for Studies on Asbestos and other Toxic Particulates “G. Scansetti”, University of Turin, Turin, Italy; 25 Department of Internal Medicine, University of Genoa and IRCSS AOU San Martino-IST-Istituto Nazionale per la Ricerca sul Cancro, Genoa, Italy; MOE Key Laboratory of Environment and Health, School of Public Health, Tongji Medical College, Huazhong University of Science and Technology, China

## Abstract

Asbestos exposure is the main risk factor for malignant pleural mesothelioma (MPM), a rare aggressive tumor. Nevertheless, only 5–17% of those exposed to asbestos develop MPM, suggesting the involvement of other environmental and genetic risk factors.

To identify the genetic risk factors that may contribute to the development of MPM, we conducted a genome-wide association study (GWAS; 370,000 genotyped SNPs, 5 million imputed SNPs) in Italy, among 407 MPM cases and 389 controls with a complete history of asbestos exposure. A replication study was also undertaken and included 428 MPM cases and 1269 controls from Australia.

Although no single marker reached the genome-wide significance threshold, several associations were supported by haplotype-, chromosomal region-, gene- and gene-ontology process-based analyses. Most of these SNPs were located in regions reported to harbor aberrant alterations in mesothelioma (*SLC7A14*, *THRB*, *CEBP350*, *ADAMTS2*, *ETV1*, *PVT1* and *MMP14* genes), causing at most a 2–3-fold increase in MPM risk. The Australian replication study showed significant associations in five of these chromosomal regions (3q26.2, 4q32.1, 7p22.2, 14q11.2, 15q14).

Multivariate analysis suggested an independent contribution of 10 genetic variants, with an Area Under the ROC Curve (AUC) of 0.76 when only exposure and covariates were included in the model, and of 0.86 when the genetic component was also included, with a substantial increase of asbestos exposure risk estimation (odds ratio, OR: 45.28, 95% confidence interval, CI: 21.52–95.28).

These results showed that genetic risk factors may play an additional role in the development of MPM, and that these should be taken into account to better estimate individual MPM risk in individuals who have been exposed to asbestos.

## Introduction

Malignant pleural mesothelioma (MPM) is a rare, aggressive tumor that generally causes death within 2 years. The only clearly established risk factors for MPM are asbestos exposure, and exposure to erionite, other mineral fibers and x-ray for medical purposes [1]. Asbestos fibers retained in the lung and pleura may be carcinogenic, either through direct mechanical or biochemical effects, or through the activation of inflammatory cells. Persistent inflammation can induce chronic oxidative stress, genotoxic lesions, chromosomal aberrations and epigenetic alterations [2], [3]. Asbestos fibers may also interfere with chromosome segregation and mitosis [4].

Although asbestos has been banned in many Western countries, it is still used in several parts of the world, and some developing countries are actually increasing the industrial use of asbestos, as well as its production and importation [5], [6], [7]. In Western Europe, over 5,000 people with MPM die each year [8], [9], [10], [11]. Considering the long median latency period between initial asbestos exposure and MPM diagnosis [12], [13], MPM incidence is expected to peak around 2020 in Western countries [9], [14], [15].

Only 5%–17% of individuals heavily exposed to asbestos develop MPM [8], suggesting a genetic component in the etiology of the disease, which is also supported by reports of familial clustering [8], [16], [17], [18] and candidate-gene association studies [8], [11]. Dominant mutations in the *BAP1* (BRCA1-associated protein 1) gene were recently reported to cause a new, rare cancer-prone syndrome that renders the individual susceptible to mesothelioma and melanoma, among others [19].

The aim of this study was to identify genetic risk factors that might contribute to the development of MPM. To this end, we performed a GWAS in an Italian study sample of 407 MPM cases and 389 healthy controls, and a replication study in an Australian study sample of 428 MPM cases and 1269 controls.

## Results

**Figure 1:**
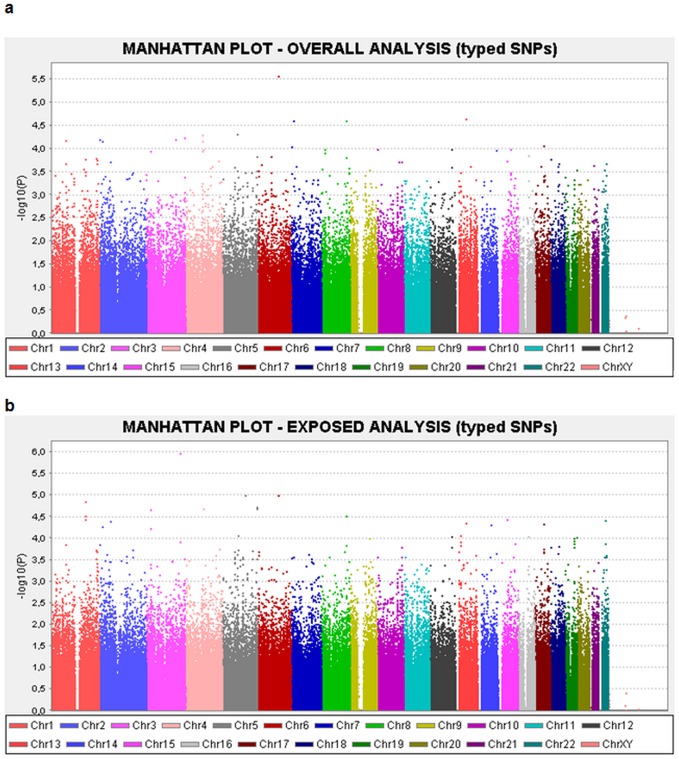
Manhattan plot of genotyped SNPs from logistic additive model. A) all samples, B) exposed samples.

The general characteristics of the Italian study sample, after quality controls (QC), are reported in Table 1 (392 MPM cases and 367 controls; 540 males, 219 females). A total of 330,879 SNPs were included in the analyses. The principal component analysis (PCA) (Figure S1) showed population stratification with two distinct clusters, which was further confirmed by K-mean analysis (data not shown). After correction of the regression analyses by PCA-cluster, the λ inflation factor was <1.03 for both the overall and the exposed-only samples (Quantile- Quantile, QQ plots, Figure S2). Manhattan plots of the two-sided logistic regression analyses (per allele additive model) are also reported (Figure 1).

**Table 1:** Summary statistics of all the subjects included in the Italian GWAS.

	CASALE M.		TURIN		GENOA		ALL SAMPLE	
	Cases	Controls	Cases	Controls	Cases	Controls	Cases	Controls
	N (%)	N (%)	N (%)	N (%)	N (%)	N (%)	N (%)	N (%)
Eligible	241 (48.88)	252 (51.12)	91 (61.9)	56 (38.1)	75 (48.08)	81 (51.92)	407 (51.13)	389 (48.87)
After QC filtering	230 (49.25)	237 (50.75)	89 (61.81)	55 (38.19)	73 (49.32)	75 (50.68)	392 (51.65)	367 (48.35)
**GENDER**								
**Males**	155 (67.39)	162 (68.35)	62 (69.66)	38 (69.09)	67 (91.78)	56 (74.67)	284 (72.45)	256 (69.75)
**Females**	75 (32.61)	75 (31.65)	27 (30.34)	17 (30.91)	6 (8.22)	19 (25.33)	108 (27.55)	111 (30.25)
**BIRTH PLACE**								
**North Italy**	204 (90.27)	186 (78.81)	62 (69.66)	35 (63.64)	54 (78.26)	53 (76.81)	320 (83,33)	274 (76,11)
**Center Italy**	6 (2.65)	12 (5.08)	5 (5.62)	1 (1.82)	8 (11.59)	5 (7.25)	19 (4,95)	18 (5)
**South Italy**	14 (6.19)	33 (13.98)	16 (17.98)	17 (30.91)	4 (5.8)	4 (5.8)	34 (8.85)	54 (15)
**Sardinia**	0 (0)	2 (0.85)	3 (3.37)	2 (3.64)	1 (1.45)	3 (4.35)	4 (1.04)	7 (1.94)
**Other Caucasians**	2 (0.88)	3 (1.27)	3 (3.37)	0 (0)	2 (2.9)	4 (5.8)	7 (1.82)	7 (1.94)
**ASBESTOS EXPOSURE**								
**Non exposed**	4 (2.06)	54 (22.78)	3 (3.37)	18 (32.73)	10 (13.7)	41 (54.67)	17 (4.87)	113 (30.79)
**Medium exposed**	106 (54.64)	103 (43.46)	33 (37.08)	25 (45.45)	7 (9.59)	22 (29.33)	146 (41.01)	150 (40.87)
**High exposed**	84 (43.3)	80 (33.76)	53 (59.55)	12 (21.82)	56 (76.71)	12 (16)	193 (54.21)	104 (28.34)
**Age (mean±s.e.)**	66.46±10.81	66.42±12.26	68.53±9.28	68.70±7.69	64.16±13.70	63.44±14.47	66.5±11.01	66.12±12.06

The genotyped SNPs with the highest significance levels are listed in Table 2. The imputed SNPs with the highest significance levels are listed in Table S1. Nine intragenic SNPs (7 genotyped and 2 imputed) were located in genes. When analyzing these nine genes in a Gene Set Enrichment Analysis (GSEA, File S1), significant enrichment involving *MMP14* and *ADAMTS2* was shown for gene-ontology (GO, File S1) biological processes including lung development (*P* = 0.0087), respiratory tube development (*P* = 0.0087), respiratory system development (*P* = 0.0087), metalloendopeptidase activity (*P* = 0.0140), and metallopeptidase activity (*P* = 0.0210) (Table S2).

**Table 2:** Italian top 12 genotyped SNP list (2-tailed logistic regression, n = 759 overall, n = 593 exposed only).

CHR Location	SNP	Ref. Allele	OR (95% CI)	P	Typed	Gene Name	Left Gene	Right Gene	Group
6q21	rs742109	A	0.55(0.43–0.71)	2.70×10^−6^	Genotyped		*PRDM1*	*ATG5*	OVERALL
3q26.2	rs7632718	A	1.83(1.42–2.37)	3.71×10^−6^	Genotyped	*SLC7A14*, *CLDN11*	*CLDN11*	*RPL22L1*	EXPOSED
3p24.2	rs9833191	C	0.54(0.41–0.71)	7.67×10^−6^	Genotyped	*THRB*	*NR1D2*	*MIR4792*	EXPOSED
5q23.1	rs1508805	A	1.85(1.41–2.44)	1.04×10^−5^	Genotyped		*PRR16*	*FTMT*	EXPOSED
1q25.2	rs2501618	A	2.18(1.53–3.10)	1.49×10^−5^	Genotyped	*CEP350*	*TOR1AIP1*	*QSOX1*	EXPOSED
5q35.3	rs4701085	G	1.84(1.39–2.44)	1.93×10^−5^	Genotyped	*ADAMTS2*	*ZNF354C*	*AX747985*	EXPOSED
4q22.1	rs4290865	A	1.98(1.44–2.71)	2.16×10^−5^	Genotyped		*FAM190A*	*GRID2*	EXPOSED
13q14.3	rs9536579	A	0.54(0.40–0.72)	2.33×10^−5^	Genotyped		*OLFM5*	*MIR1297*	OVERALL
7p21.2	rs3801094	A	1.75(1.35–2.27)	2.52×10^−5^	Genotyped	*ETV1*	*ARL4A*	*DGKB*	OVERALL
8q24.21	rs7841347	A	0.60(0.47–0.76)	2.60×10^−5^	Genotyped	*PVT1*	*MYC*	*TMEM75*	OVERALL
15q21.1	rs10519201	A	2.36(1.57–3.56)	3.82×10^−5^	Genotyped	*SHC4*	*EID1*	*SECISBP2L*	EXPOSED
22q12.3	rs5756444	G	0.60(0.47–0.76)	3.95×10^−5^	Genotyped		*CSF2RB2*	*C22orf33/TEX33*	EXPOSED

When the GSEA (File S1) was extended to SNPs with a significance level of *P*≤10^−4^ in the regression analysis (additive model, 201 genes), another metallopeptidase, namely *MMP8*, was included in the gene list, further reinforcing the putative role of the metalloendopeptidase pathway in MPM.

Haplotype association was investigated in the Italian study sample for the 20 genes/chromosomal regions with the highest significance levels. The most significant haplotype associations were found in the chromosomal region 3p24.2, where the *THRB* gene is located (*P* = 2.04×10^−7^), and in 19q13.42 (*P* = 7.02×1 0^−7^) (Table S3), strengthening the importance of these chromosomal regions.

**Figure 2:**
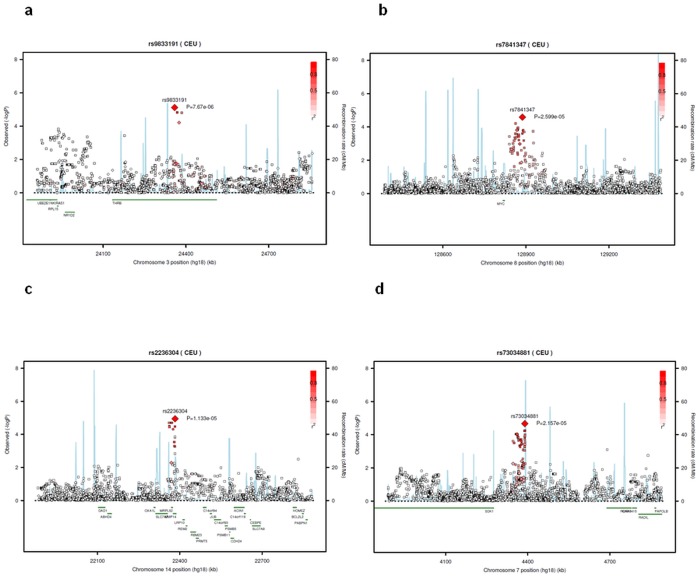
Regional association plots for 4 of the most consistent chromosome regions. a. 3p24.2, b. 8q24.21, c. 14q11.2, d. 7p22.2. Consistency was based on haplotype, gene-, region- and pathway analysis. Each SNP is plotted with respect to its chromosomal location (*x* axis) and its log_10_ transformed *P* value (*y* axis on the left) for associations with MPM. The tall blue spikes indicate the recombination rate (*y* axis on the right) at that region of the chromosome. The red-outlined diamond indicate the index SNP and other diamond indicate the genotyped SNPs, the squares indicate imputed SNPs using as reference 1000 Genomes Pilot 1 CEU population. LD values were calculated only on our control population.

Seven chromosomal regions were significantly associated with MPM in the region-based analysis (*P*<0.0025, Table 3, Figure 2, Figure S3) [20]. The gene-based analysis confirmed the significance of the *THRB* gene (*P* = 2.29×10^−5^) and showed a borderline significance for the *PVT1* gene (*P* = 0.02) (Table 3). Finally, the regional GO (File S1) process-based analysis supported the involvement of the metalloendopeptidase and metallopeptidase GO (File S1) processes (Table 3, *P* = 0.0005 and 0.0039, respectively).

**Table 3:** Region-, Gene- and GO process-based analysis on top SNPs (1-tailed binomial test, n = 759, alpha 0.0025, alpha = 0.01, alpha = 0.025, respectively).

Region/Gene/GO processes based	Cytogenetic Band	Position (from - to)	Number of SNPs	Significant SNPs	P
-	1q25.2	(178192161–178267165)	5	4	8.31×10^−4^
-	3p24.2	(24311166–24397755)	17	7	3.86×10^−4^
-	3q26.2	(171668688–171738200)	12	6	9.47×10^−5^
-	4q22.1	(92842088–92925574)	11	3	0.05
-	4q32.1	(160680345–160763147)	11	3	0.04
-	5q23.1	(120950796–121034917)	11	3	0.08
-	5q35.2	(173515657–173599925)	16	4	7.23×10^−3^
-	5q35.3	(178559043–178654962)	19	5	0.01
-	6q21	(106656091–106738553)	18	5	8.00×10^−3^
-	7p21.2	(13877273–13974190)	20	6	4.36×10^−3^
-	7p22.2	(4339181–4436371)	17	9	5.96×10^−5^
-	8q24.21	(128837336–128935399)	7	6	1.04×10^−4^
-	9p24.1	(5363441–5453988)	12	5	0.02
-	12q23.3	(107375486–107461372)	13	7	5.78×10^−5^
-	13q14.3	(53429288–53513774)	12	4	0.02
-	14q11.2	(22334110–22425388)	13	2	0.14
-	15q14	(34381353–34470568)	13	5	2.04×10^−3^
-	15q21.1	(46959609–47047893)	18	2	0.23
-	19q13.42	(59189856–59266559)	9	1	0.47
-	22q12.3	(35660028–35754794)	19	5	0.03
*CEP350*	1q25.2	(179933906–180093734)	17	2	0.31
*THRB*	3p24.2	(24162088–24541232)	54	15	2.29×10^−5^
*SLC7A14*	3q26.2	(170167538–171715102)	13	2	0.16
*SDK1*	7p22.2	(3341374–4303003)	90	5	0.61
*PVT1*	8q24.21	(128808953–129119976)	34	7	0.02
*METALLOENDOPEPTIDASE*	-	-	197	19	4.65×10^−3^
*METALLOPEPTIDASE*	-	-	470	32	0.04

**Figure 3:**
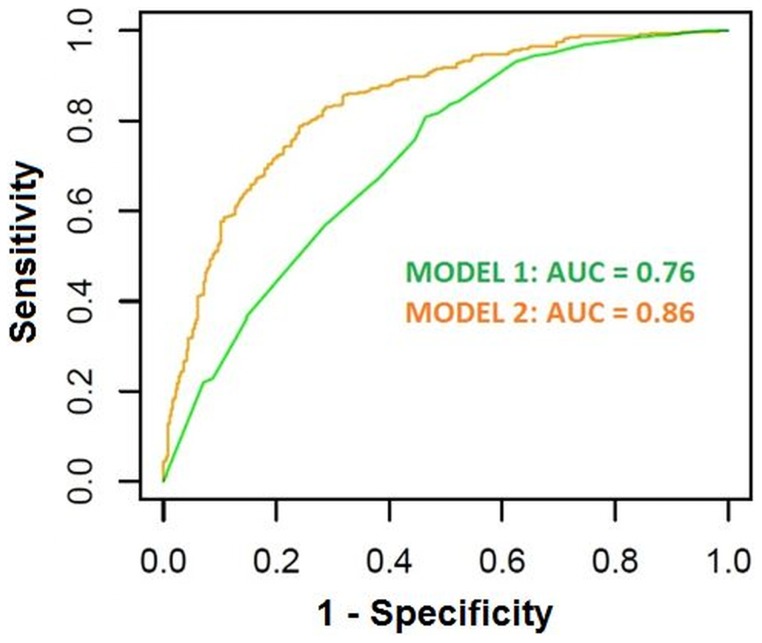
Receiver Operating Curves (ROC) for the two multivariate models including asbestos exposure 1) without and 2) with the 10 most robust and significant genetic variants.

We detected a substantial improvement in accuracy comparing the first multivariate model, which used asbestos exposure as a predictor and adjusted for demographic covariates, with the second one, which also included 10 selected SNPs with independent effects (Table 4). The average Akaike Information Criterion (AIC) and area under ROC curve (AUC) across 10,000 random splits of the entire Italian study sample were 871.34 and 0.76 for the first model, and 730.27 and 0.86 for the second model, respectively (Figure 3, Table 4). The analysis stratified by center (Casale Monferrato versus Turin-Genoa) confirmed the stability of the risk estimates and 95% CIs (data not shown).

**Table 4:** Nested multivariate logistic regression models: 1) model 1, without genetic component; 2) model 2, with genetic component.

	MODEL 1				MODEL 2				
	OR	OR_95L	OR_95U	P	OR	OR_95L	OR_95U	P	GENETIC MODEL
LOW vs NO EXPOSURE	8.01	4.41	14.54	8.52×10^−12^	15.31	7.78	30.14	2.86×10^−15^	-
HIGH vs NO EXPOSURE	17.33	9.28	32.37	<2×10^−16^	45.28	21.52	95.28	<2×10^−16^	-
CLUSTER 2 vs 1	1.76	1.1	2.79	1.74×10^−02^	2.21	1.29	3.79	4.09×10^−03^	-
rs2501618	-	-	-	-	2.23	1.47	3.37	1.52×10^−04^	dominant
rs9833191	-	-	-	-	0.55	0.41	0.73	4.39×10^−05^	additive
rs7632718	-	-	-	-	1.85	1.41	2.42	9.07×10^−06^	additive
rs4701085	-	-	-	-	2.05	1.41	2.97	1.75×10^−04^	dominant
rs73034881	-	-	-	-	0.44	0.29	0.67	1.12×10^−04^	additive
rs3801094	-	-	-	-	1.86	1.39	2.48	2.78×10^−05^	additive
rs7841347	-	-	-	-	0.51	0.39	0.67	1.56×10^−06^	additive
rs10815216	-	-	-	-	0.41	0.27	0.60	8.53×10^−06^	dominant
rs2236304	-	-	-	-	1.72	1.19	2.51	4.39×10^−03^	dominant
rs7178364	-	-	-	-	0.45	0.28	0.71	5.66×10^−04^	dominant

* adjusted for age, gender and center of recruitment.

MODEL 1: AIC = 871.3, AUC = 0.76.

MODEL 2: AIC = 730.27, AUC = 0.86.

The first multivariate model confirmed asbestos exposure as the main risk factor for MPM (high exposure: OR 17.33, 95% CI 9.28–32.37, *P*<2×10^−16^; low exposure: OR 8.01, 95% CI 4.41–14.54, *P* = 8.52×10^−12^) (Table 4). The second model, which included the genetic component, showed that the 10 selected SNPs had an independent contribution to MPM risk (Table 4), and also increased the estimate for the effect of asbestos exposure (high exposure: OR 45.28, 95% CI 21.52–95.28, *P*<2×10^−16^; low exposure: OR 15.31, 95% CI 7.78–30.14, *P* = 2×10^−15^).

### SNP validation and replication

The Italian and Australian study samples showed a marked degree of heterogeneity (I^2^ statistics, range 0.62–0.97) [21] (Table S5). None of the 12 genotyped SNPs with the highest significance levels in the Italian study were found in the Australian replication study (Table S4), and nor of these were confirmed by the meta-analysis (Table S5). Nevertheless, when a regional analysis was performed in the Australian study sample, we found significant associations in five chromosomal regions (3q26.2, 4q32.1, 7p22.2, 14q11.2, 15q14) that have reported to be altered in mesothelioma (Table 5) [20].

**Table 5:** Regional replication of Italian top signals in the Australian study for 5 out of the 20 regions.

Cytogenetic Band	BP_start^a^	BP_end^a^	p Binomial test^b^	p Binomial test^c^	Meta-analysis
3q26.2	171668688	171738200	9.47338E-05	0.01643691	1.61×10^−5^
4q32.1	160680345	160763147	0.042137914	0.000649	3.15×10^−4^
7p22.2	4339181	4436371	5.95584E-05	0.01403811	1.26×10^−5^
14q11.2	22334110	22425388	0.139471486	0.00100497	1.38×10^−3^
15q14	34381353	34470568	0.002040183	0.01305659	3.07×10^−4^

(1-tailed binomial test and meta-analysis).

a NCBI36/hg18.

b Italian study.

c Australian study.

### Gene expression analysis in blood and in normal pleural tissue

**Figure 4:**
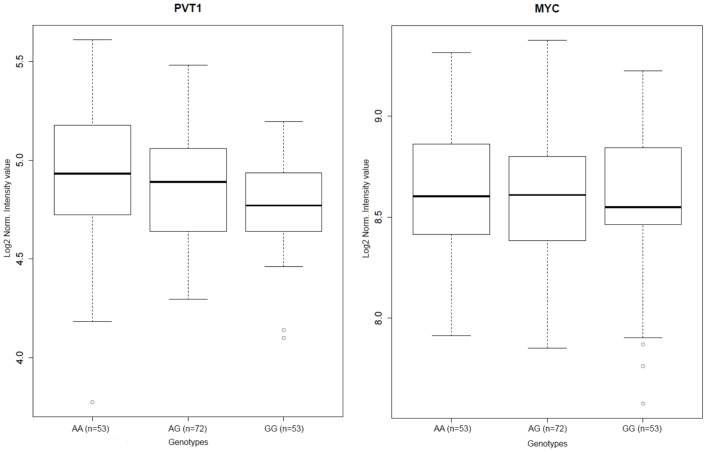
eQTL: *PVT1* and *MYC* gene-expression levels in blood cells across rs78941347 genotypes.

Gene expression analysis on lymphocytes from Italian healthy subjects (Text S1) showed a possible expression Quantitative Trait Locus (eQTL) for the *PVT1* (rs7841347) gene (non-parametric Kruskal-Wallis test *P*<0.001) (Figure 4). However, expression analysis from Italian healthy subjects pleural tissue stratified by *PVT1* rs7841347 genotypes did not show any gradient, although a statistically significant difference (*P* = 0.01) was found (Figure S4). Published expression data [22] (Text S1) confirmed the dysregulation of *MMP14*, *THRB* and *MYC* genes in MPM, supporting our results.

### SNP predictive functional analysis

Using the GenomePipe tool, none of the SNPs with the highest significance levels included in the present analysis might predict damage, nor were they located in a regulatory or splicing site. Even when SNPs in Linkage Disequilibrium (LD) with our top SNPs (LD≥0.8 as measured by pairwise r^2^) were included in the analysis no evidence of functional properties of the proxy SNPs was found. LD refers to two different populations, i.e. HapMap TSI from Tuscany (Italy) and CEU (HapMap3, File S1), for a total of 33 and 72 SNPs respectively.

## Discussion

In order to identify genetic risk factors that might contribute to the development of MPM, we performed a GWAS on 407 Italian MPM cases and 389 controls.

We performed an independent replication study in an Australian sample, which included 428 MPM cases (Genetic Understanding of Asbestos-Related Disease, GUARD, study) and 1,269 controls (Busselton Health Study, BHS).

Among the top SNPs identified in our Italian study sample, there were several genes previously reported to be involved in MPM or other cancer types, as well as chromosomal regions reported to be altered in MPM [20].

Although no single SNP replicated in the Australian sample, probably due to the high genetic heterogeneity between the two studies, regional analyses showed significant signals in 5 of the chromosomal regions where the Italian top SNPs are located. The chromosomal region 7p22.2 found in the replication study includes the *SDK1* [23] and *FOXK1* [24] genes. Interestingly, FOXK1 has been reported to interact with BAP1 [25], which was recently found to be mutated in mesothelioma [19]. Chromosomal region 7p22 is located in a fragile sequence (*FRA7B*) containing two miRNA genes (mir589 and mir339) and three large genes (*SDK1*, *THSD7A*, *MAD1L1*), and is highly prone to gaps and breaks in several cancers [23].

Another Italian genotyped top-signal (rs7632718) is located in the *SLC7A14* (solute carrier family 7 member 14) gene, which lies on 3q26.2, which was one of the replicating regions in the Australian study. Although no link with MPM has been previously reported for *SLC7A14*, a chromosomal gain has been described in this region [20], suggesting a possible involvement of other genes in MPM.

The *PVT1* (Pvt1 oncogene (non-protein coding)) gene is involved in several types of cancer [26], [27], [28], [29], [30]. It is located in a large (>300 kb) locus downstream of *MYC* (53 Kb apart) on chromosomal region 8q24. The *PVT1* locus produces a wide variety of spliced non-coding RNAs as well as a cluster of six annotated miRNAs: miR-1204, miR-1205, miR-1206, miR-1207-5p, miR-1207-3p, and miR-1208 [31], [32]. *PVT1* was proposed to regulate *c-Myc* gene transcription over a long distance [33]. A functional variant (rs378854) in chromosomal region 8q24 that modulates *PVT1* expression has been associated with prostate cancer [34]. *In vitro*, the rs378854-G allele has been associated with reduced binding of the transcription factor YY1, a putative tumor suppressor, and with repressed global transcription in prostate cancer [33]. The regulation of this chromosomal region is very complex, as is suggested by the association of several SNPs with different cancer types [35], and involves miRNA, lincRNA and other epigenetic regulations [36].

The gene-expression analysis on lymphocytes from Italian healthy subjects showed a possible eQTL for *PVT1*. Functional studies are needed to clarify the link between *PVT1*-associated SNPs, gene expression regulation and cancer risk taking into account that in our study *PVT1* seems to act only at an early stage of carcinogenesis as its deregulation has not been observed at later stages in tumor tissue [22].

Two other genes that have been reported to be dysregulated in MPM, are *THRB* and *MMP14* [22], [37]. *THRB* encodes for thyroid hormone receptor beta (TRβ), which could function as a tumor suppressor. Cell-based studies and xenograft models have demonstrated that TRβ is a suppressor of ras-mediated cell proliferation, transformation, and tumorigenesis [38]. Moreover, TRβ disrupts mitogenic growth factors by suppressing the activation of extracellular signal-regulated kinases and phosphatidylinositol 3-kinase signaling pathways to suppress tumor cell invasiveness and metastasis [39], [40]. *THRB* is located about 28 Mb telomeric to the *BAP1* gene, which is mutated in MPM [19]. A down-regulation of *THRB* has been documented in MPM versus parietal pleura [41] and it is frequently methylated/deleted in non-squamous-cell lung cancer [42].

*MMP14* (matrix metallopeptidase 14) has been reported to influence overall survival in MPM cases [37], and was significantly highlighted in our enrichment analysis, together with ADAMTS2, because of their metalloendopeptidase and metallopeptidase activities. The matrix metalloproteinases are a family of zinc-containing enzymes with proteolytic activity against a wide range of extracellular proteins. Extracellular matrix proteases are involved in several steps of cancer development and progression, including angiogenesis and metastasis.

Some of the SNPs with highest significance levels were located in the genes: *CEP350*, *ETV1* and *SHC4*. Although they have not been directly associated with MPM, their involvement in several cancer types has been described [43], [44], [45], suggesting the necessity to further investigate their possible role in MPM pathogenesis. Considering the closest flanking genes of intergenic SNPs, the following are noteworthy and could contribute to the carcinogenic process, as has been reported for other cancer types: *PRDM1* [46], *ATG5* [47], *MYC* [48], *EID* [49], *RLN1* [50], *CD274* [51].

Although our sample size is clearly a limitation for a GWAS, the Italian and the Australian study samples are, to the best of our knowledge, the largest MPM series with available DNA, as mesothelioma is a very rare cancer. A further limitation of GWAS is that they do not take into account rare variants. The availability of methods for complete genome sequencing (and the decrease of the sequencing costs) will allow to circumvent the problem linked to the identification of rare variants, whose involvement should be better investigated in future studies.

The negative replication of the Italian top SNPs in the Australian study should be revised on the basis of the following considerations: i) the two studies had a marked degree of heterogeneity as shown by the I^2^ statistics; ii) no exposure assessment was available for the Australian control group. Notwithstanding these discrepancies, we observed an intriguing significant regional replication in the Australian study for 5 out of 20 Italian top signals.

Most of the top-signals we identified were located in chromosomal regions reported to harbor aberrant alterations in mesothelioma, and cause an at most 2–3 fold increase in MPM risk.

Moreover, asbestos exposure in our study was associated with a remarkable increase in MPM risk, which became even more evident when the contribution of genetic factors was taken into account, with a significant improvement of asbestos exposure risk estimation.

In conclusion, our results support the complementary role of genetic background in asbestos-related carcinogenesis of the pleura, indicating that genetic risk factors should be taken into account to understand MPM physiopathology, and to better define the MPM risk profile of people with a high exposure to asbestos.

## Methods

### Ethics statement

All MPM cases reported on in the present report gave written informed consent. This study was performed according to the principles of the Declaration of Helsinki and in agreement with ethical requirements. Approval was obtained from the Istituto Nazionale per la Ricerca sul Cancro Ethics Committee for the studies in Genoa and La Spezia, and from the Human Genetics Foundation (HuGeF) Ethics Committee for the studies in Casale Monferrato and Turin. The Australian replication study was specifically approved by the Human Research Ethics Committee of the University of Western Australia.

### Italian study sample

The Italian study sample is composed of MPM cases and controls from cities located in Northern Italy: Casale Monferrato and Turin in the Piedmont Region, and Genoa and La Spezia in the Liguria Region (Table 1; details in Text S1). The study in Casale Monferrato was a population-based MPM case-control study [52], and included 241 MPM patients and 252 population controls of Italian nationality and Caucasian ethnicity. The study in Turin was a hospital-based MPM case-control study [11], and consisted of 91 MPM patients and 56 controls of Italian nationality and Caucasian ethnicity. The hospital-based study in Genoa and La Spezia included 75 incident MPM cases [53]. Controls are 81 healthy subjects or patients hospitalized for non-neoplastic/non-respiratory conditions.

All the three of the above-mentioned Italian studies were registry-based and therefore no selection criteria were applied to MPM cases; they needed only to be residing in the study area at the time of diagnosis. Only cases with a pathological diagnosis (based on histology or cytology with confirmatory immunohistochemical staining) were eligible for inclusion in the present analysis. Study periods in the Italian studies were different (Casale Monferrato: January 2001 to December 2006; Turin: January 2004 to October 2008; Genoa and La Spezia: April 1996 to February 2006 for cases and February 1997 and November 2006 for controls). For practical reasons, the study in Turin was limited to cases admitted to the main metropolitan hospitals.

Asbestos exposure was carefully assessed in all the Italian cases and controls. After reviewing individual occupational histories, asbestos exposure was reclassified for the overall sample by the same expert (D.M.) as “no/unlikely” (no acknowledged occupational or environmental exposure), “low” (low exposure probability, or definite low exposure), and “high” (definite and high exposure; asbestos-cement and asbestos-textile workers, insulators, shipyard workers and dockers).

### Australian replication study

Australian MPM cases were part of the GUARD study, which consisted of individuals who had been exposed to asbestos and diagnosed with MPM (n = 428) and who attended a hospital clinic in Perth, Western Australia between 1988 and 2010 [54]. DNA samples and clinical data from these individuals were obtained and MPM diagnosis was confirmed after pathological, radiological and clinical review with confirmation from respective cancer registries in Western Australia (Western Australia Mesothelioma Registry) and Queensland.

The GUARD study subjects are primarily male (88.8%) with an average age of 67±10.3 years. Most BHS study subjects are female (57.4%) and the average age is 54±17.2 years. Control samples (n = 1,269), with no information on asbestos exposure, were obtained from the population-based BHS [55]. MPM cases were excluded after genotyping if they were: related to another individual, had a low call GWAS rate (<97%), were not Caucasian/European based on principal component analysis, had ambiguous sex, or had low heterozygosity compared to the rest of the sample.

### SNP genotyping

Whole-genome genotyping was done on a HumanCNV370-Quad BeadChip (Illumina Inc., San Diego, CA, USA) for 716 samples. The remaining 80 samples were tested on a Human610-Quad (which includes 100% of the HumanCNV370 BeadChip SNPs) as the HumanCNV370-Quad had been discontinued. Genotypes were assessed by GenomeStudio V2011.1(Illumina Inc., San Diego, CA). The 12 most significant SNPs from the Italian studyS were individually genotyped in the Australian replication study with a 5′-nuclease assay (AppliedBiosystems, CA, USA).

### Statistical analysis

#### Genotyping quality controls

A cut-off a genotyping call rate of 0.98 was set, leading to the exclusion of 18 study subjects. SIdentity By Descent (IBD) estimation using the Identity By State (IBS) distance was used to check genotypic identity or relatedness among subjects (PLINK software [56], File S1). Subjects with IBD≥0.05 (n = 16) were considered consanguineous and excluded from further analyses. We additionally excluded three samples with an X chromosome inbreeding homozygosity estimate of about 0.5. Thirty-seven subjects (4.64%) were removed from the analysis, leaving 759 subjects (392 cases and 367 controls).

SNPs with minor allele frequency <1% (n = 15,252), those having >0.05 missing genotypes (n = 11,535) and those deviating from Hardy-Weinberg equilibrium (HWE) in the control population (*P*<0.001, n = 1,157) were excluded from the analysis, for a final study data-set of 330,879 SNPs, which were analyzed for their potential association with mesothelioma.

#### Population structure and association analysis

The population structure was investigated by PCA (PLINK Software, File S1, Covariance Method [57]). A new discrete covariate was defined by the two principal components (Figure S1), and was included in the following logistic regression analysis. PCA results were further confirmed by the K-means clustering analysis [58] (data not shown). The effective removal of any population structure bias was checked by the λ-inflation factor parameter [59] (Figure S2).

We tested for 330,879 SNPs for their association with mesothelioma by 2-sided logistic regression analysis on a per-allele additive model after adjusting for age, gender, PCA cluster, center of recruitment and exposure level, both in the overall Italian sample (n = 759) and among exposed-only Italian subjects (n = 593) (high and low exposure). After Bonferroni correction, we considered alpha = 1.51×10^−7^ (0.05/330879) as a threshold of significance. The analyses were performed with PLINKv1.07 (File S1) [56] and Rv2.10.1 [60] software. The software Impute.v2 [61], [62] was used to impute 5,333,982 SNPs, using the 1000 genomes (http://www.1000genomes.org/) and HapMap3 (File S1) genotype panels as reference datasets.

Haplotypes (Table S3) within the chromosomal regions where the most significant SNPs were located (considering sliding windows from 2 to 10 SNPs; PLINK Software, File S1) were also tested for any association with MPM in the overall Italian sample.

#### Meta-analysis and replication

A meta-analysis of the Italian-study top 12 genotyped SNPs was done on data from the whole genome genotyping (Human610-Quad BeadChip, Illumina) of 428 cases and 1269 Australian controls of European descent (GWAMA software, File S1 [63]). A random-effects model was used due to the presence of genetic heterogeneity (I^2^ statistic [21] >50%; Table S5).

#### Multivariate analysis

The cumulative effect of the SNPs with highest significance levels was investigated by two-sided multivariate logistic regression analysis, comparing the prediction accuracy of two models: the first considering asbestos exposure as a predictor and adjusting for demographic covariates (recruitment center, gender, age, geographical cluster), and the second identical to the first, but also including the genetic component (genotypes). SNPs included in the second multivariate model were selected among the top 20 markers (12 genotyped and 8 imputed), excluding 4 SNPs (rs4290865, rs1354252, rs1072577, rs10519201) because of negative internal replication between Casale Monferrato and pooled Turin-Genoa studies, and 6 SNPs (rs742109, rs1508805, rs9536579, rs5756444, rs6897549, rs71365421) because they did not replicate in the Australian study on the regional analysis and were not intragenic.

An internal validation of the two models was done by randomly splitting the overall Italian sample in two groups 10,000 times, each time performing a two-sided logistic regression in the first group and verifying the accuracy of estimation in the second group. The average AIC under 10,000 permutations and AUC were used as measures of the fit and the prediction power of the two models.

#### Gene-region enrichment and SNP functional prediction analyses

A GSEA (File S1) [64] was performed on the genes in which the top SNPs are located (9 genes out of 20 signals): *PVT1* (gene ID 5820), *CEP350* (ID 9857), *THRB* (ID 7068), *ETV1* (ID 2115), *C9orf46* (also known as *PLGRKT*; ID 55848), *MMP14* (ID 4323), *ADAMTS2* (ID 9509), *SLC7A14* (ID 57709), *SHC4* (ID 399694). The list was tested for over-representation using the curated Molecular Signatures Database (MSigDB) 7, specifically i) KEGG 8 (File S1), REACTOME and BioCarta pathway databases, ii) the GO (File S1) gene set 9. Gene set enrichment significance was tested by a hyper-geometric test that evaluates the distribution of overlapping genes over all genes in the gene set (Table S2).

Region-, gene- and GO (File S1) process-based analyses were also performed [65]. We investigated the occurrence of multiple signals in those genes and chromosomal regions, where the significant SNPs from the single SNP analysis are located, as well as those from genes belonging to the pathways identified by the GO (File S1) process-based analysis (Table 3).

We tested 20 candidate chromosomal regions, and five genes (*CEP350*, *THRB*, *SLC7A14*, *SDK1* and *PVT1*) for which there were enough representative SNPs genotyped, and two GSEA significant GO processes (File S1) (metalloendopeptidase activity and metallopeptidase activity). After Bonferroni correction, we adopted the following significance thresholds: alpha = 0.0025, alpha = 0.01, alpha = 0.025, for region-based, gene-based and GO (File S1) process-based analysis respectively.

Prediction of functional SNPs has been carried out with several softwares, including GenomePipe software, which is freely available at website of the National Institute of Environmental Health Sciences (http://snpinfo.niehs.nih.gov/seleGWAs.htm) and the Pupasuite3.1 software (http://pupasuite.bioinfo.cipf.es/).

#### Gene-expression analysis

The expression levels of the nine genes corresponding to the most common intragenic SNPs (Table 2) and of *MYC*, which is neighbor to *PVT1*, were examined using data from the HapMap (File S1) CEU gene-expression database, and the GenoPheno database [66], an internal database which includes genotypic, phenotypic, and gene-expression data from the peripheral blood of 120 healthy Italian volunteers (Text S1). We considered the average expression levels of probes and, when feasible, tested for differential expression among the three genotypes (Kruskal-Wallis test).

In addition, the mRNA levels of the *PVT1*, *MYC* and *THRB* genes were measured by quantitative real-time PCR in 79 normal pleural tissues from donors that underwent thoracoscopy for conditions other than MPM, who signed an informed consent form (Text S1).

## Supporting Information

Figure S1 **Principal Component Analysis (PCA) plots: first** ***vs*** **second PC.** A) Cases and controls are plotted for the overall study and for each of the three study samples (Turin, Casale Monferrato and Genoa); B) birth places (Northern, Central, Southern Italy, Sardinians and Other Caucasians) are plotted for the overall study and for each of the three study samples.
(TIFF)

Figure S2 **Supplementary** **figure 1** **:Q-Q plots for GWAS of mesothelioma in the Italian population.** This Q-Q plots are based on logistic regression allelic *P* after standard quality control. The estimated λ inflation factor was <1.03. Plot A shows the Q-Q plot for the overall Italian population, whereas Plot B refers to the exposed-only population.
(TIFF)

Figure S3 Regional association plots for additional 4 regions (a. 3q26.2, b. 4q32.1, c. 7p21.2, d. 15q14) replicating in the Australian study. Each SNP is plotted with respect to its chromosomal location (*x* axis) and its log_10_ transformed *P* value (*y* axis on the left) for associations with MPM. The tall blue spikes indicate the recombination rate (*y* axis on the right) at that region of the chromosome. The red-outlined diamond indicate the index SNP and other diamond indicate the genotyped SNPs, the squares indicate imputed SNPs using as reference 1000 Genomes Pilot 1 CEU population. LD values were calculated only on our control population
(TIFF)

Figure S4 **RT-PCR of** ***PVT1*** **and** ***MYC*** **genes-expression levels in 79 normal pleural tissues expression levels across rs78941347 genotypes.**
(TIFF)

Table S1 **Italian top 8 imputed SNP list.**
(DOCX)

Table S2 **Gene Set Enrichment Analysis.**
(DOCX)

Table S3 **Significant Haplotype Results for 3p24 and 19q13.42 regions.**
(DOCX)

Table S4 **Replication of the 12 genotyped Italian top SNPs on GUARD-BHS Study.**
(DOCX)

Table S5 **Meta-analysis of Italian and Australian studies for the top 12 genotyped Italian SNPs.**
(DOCX)

Text S1 **Supplementary Materials.**
(DOCX)

File S1 **URLs.**
(DOCX)
