# Correction: Genetic Variants Associated with Increased Risk of Malignant Pleural Mesothelioma: A Genome-Wide Association Study

**DOI:** 10.1371/journal.pone.0130109

**Published:** 2015-06-03

**Authors:** Giuseppe Matullo, Simonetta Guarrera, Marta Betti, Giovanni Fiorito, Daniela Ferrante, Floriana Voglino, Gemma Cadby, Cornelia Di Gaetano, Fabio Rosa, Alessia Russo, Ari Hirvonen, Elisabetta Casalone, Sara Tunesi, Marina Padoan, Mara Giordano, Anna Aspesi, Caterina Casadio, Francesco Ardissone, Enrico Ruffini, Pier Giacomo Betta, Roberta Libener, Roberto Guaschino, Ezio Piccolini, Monica Neri, Arthur W. B. Musk, Nicholas H. de Klerk, Jennie Hui, John Beilby, Alan L. James, Jenette Creaney, Bruce W. Robinson, Sutapa Mukherjee, Lyle J. Palmer, Dario Mirabelli, Donatella Ugolini, Stefano Bonassi, Corrado Magnani, Irma Dianzani

There are errors in Table 1. The values in the row labeled Age (mean ± s.e) are incorrect. Please see the corrected Table 1 here.

**Table 1:** Summary statistics of all the subjects included in the Italian GWAS.

	CASALE M.		TURIN		GENOA		ALL SAMPLE	
	Cases	Controls	Cases	Controls	Cases	Controls	Cases	Controls
	N (%)	N (%)	N (%)	N (%)	N (%)	N (%)	N (%)	N (%)
Eligible	241 (48.88)	252 (51.12)	91 (61.9)	56 (38.1)	75 (48.08)	81 (51.92)	407 (51.13)	389 (48.87)
After QC filtering	230 (49.25)	237 (50.75)	89 (61.81)	55 (38.19)	73 (49.32)	75 (50.68)	392 (51.65)	367 (48.35)
**GENDER**								
** Males**	155 (67.39)	162 (68.35)	62 (69.66)	38 (69.09)	67 (91.78)	56 (74.67)	284 (72.45)	256 (69.75)
** Females**	75 (32.61)	75 (31.65)	27 (30.34)	17 (30.91)	6 (8.22)	19 (25.33)	108 (27.55)	111 (30.25)
**BIRTH PLACE**								
** North Italy**	204 (90.27)	186 (78.81)	62 (69.66)	35 (63.64)	54 (78.26)	53 (76.81)	320 (83,33)	274 (76,11)
** Center Italy**	6 (2.65)	12 (5.08)	5 (5.62)	1 (1.82)	8 (11.59)	5 (7.25)	19 (4,95)	18 (5)
** South Italy**	14 (6.19)	33 (13.98)	16 (17.98)	17 (30.91)	4 (5.8)	4 (5.8)	34 (8.85)	54 (15)
** Sardinia**	0 (0)	2 (0.85)	3 (3.37)	2 (3.64)	1 (1.45)	3 (4.35)	4 (1.04)	7 (1.94)
** Other Caucasians**	2 (0.88)	3 (1.27)	3 (3.37)	0 (0)	2 (2.9)	4 (5.8)	7 (1.82)	7 (1.94)
**ASBESTOS EXPOSURE**								
** Non exposed**	4 (2.06)	54 (22.78)	3 (3.37)	18 (32.73)	10 (13.7)	41 (54.67)	17 (4.87)	113 (30.79)
** Medium exposed**	106 (54.64)	103 (43.46)	33 (37.08)	25 (45.45)	7 (9.59)	22 (29.33)	146 (41.01)	150 (40.87)
** High exposed**	84 (43.3)	80 (33.76)	53 (59.55)	12 (21.82)	56 (76.71)	12 (16)	193 (54.21)	104 (28.34)
**Age (mean±s.e.)**	67.61±11.14	63.36±11.06	68.74±8.84	68.31±8.80	69.64±9.64	58.59±15.03	68.25±10.39	63.11±12.01
