# Supplementary figures and images for: Genetic Variants Associated with Increased Risk of Malignant Pleural Mesothelioma: A Genome-Wide Association Study
Source: PLoS One. 2013 Apr 23;8(4):e61253. doi: 10.1371/journal.pone.0061253 (PMC3634031; doi:10.1371/journal.pone.0061253)

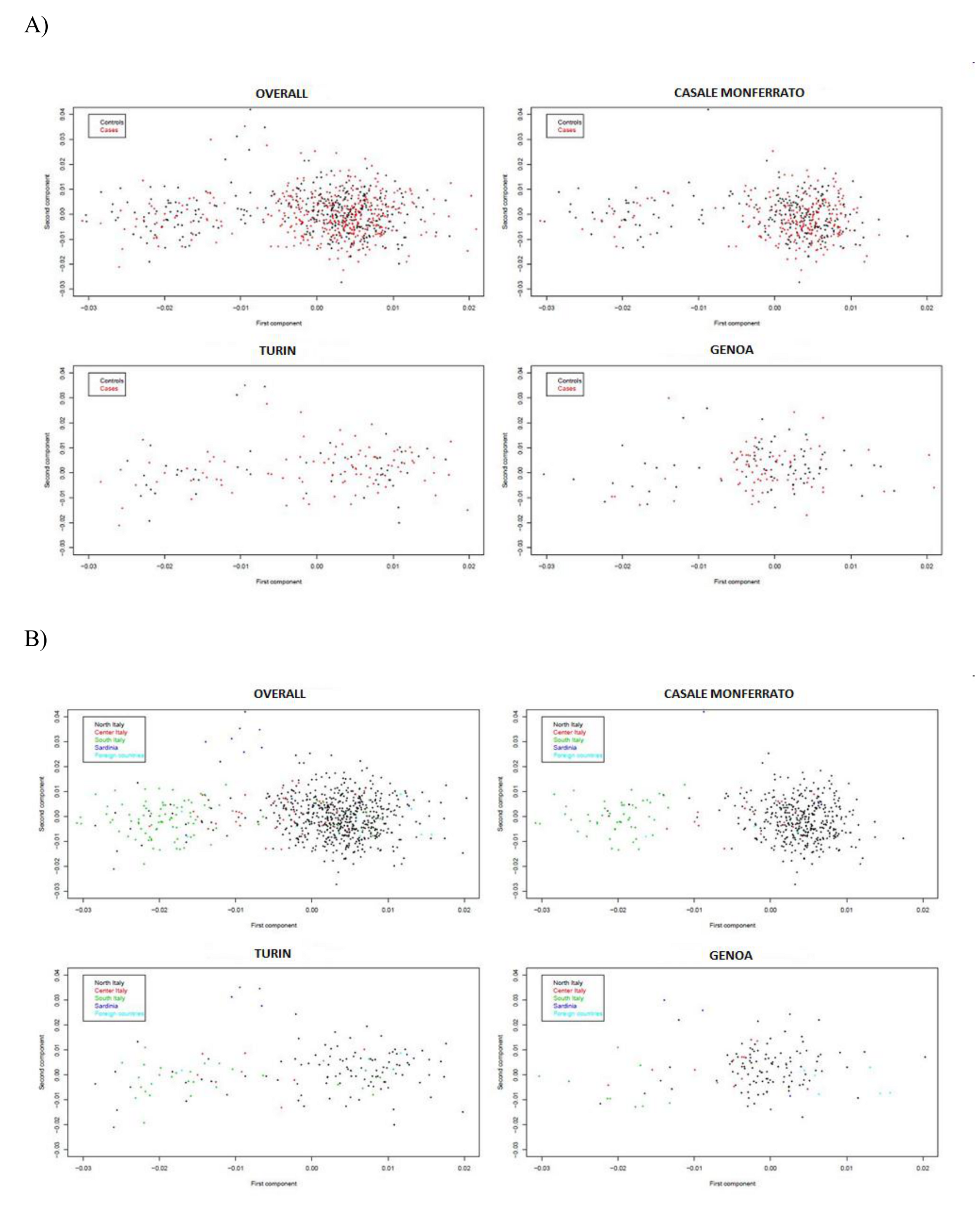

Supplement: Figure S1 — Principal Component Analysis (PCA) plots: first vs second PC. A) Cases and controls are plotted for the overall study and for each of the three study samples (Turin, Casale Monferrato and Genoa); B) birth places (Northern, Central, Southern Italy, Sardinians and Other Caucasians) are plotted for the overall study and for each of the three study samples. (TIFF) [file pone.0061253.s001.tiff]

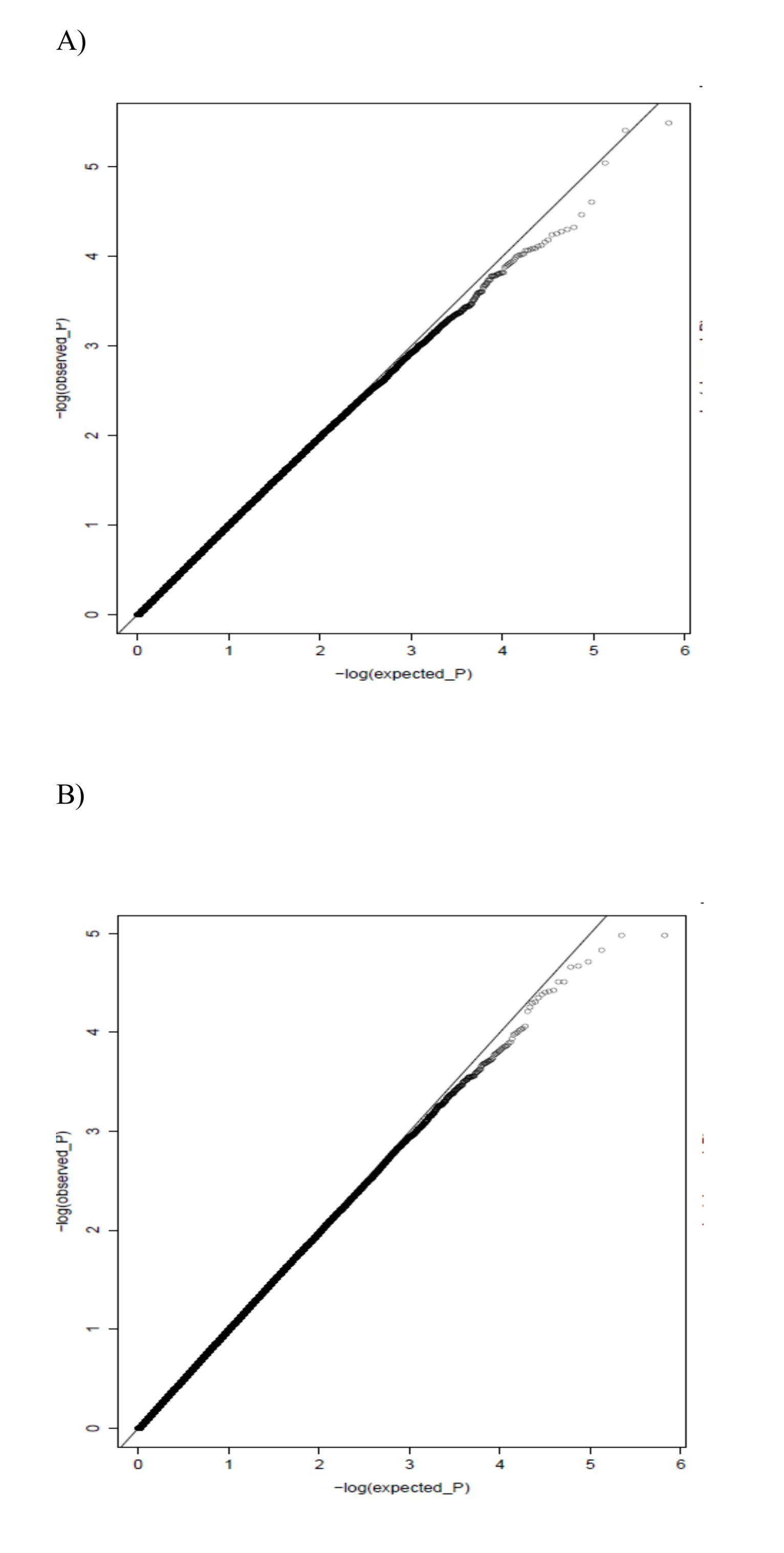

Supplement: Figure S2 — Supplementary figure 1 :Q-Q plots for GWAS of mesothelioma in the Italian population. This Q-Q plots are based on logistic regression allelic P after standard quality control. The estimated λ inflation factor was <1.03. Plot A shows the Q-Q plot for the overall Italian population, whereas Plot B refers to the exposed-only population. (TIFF) [file pone.0061253.s002.tiff]

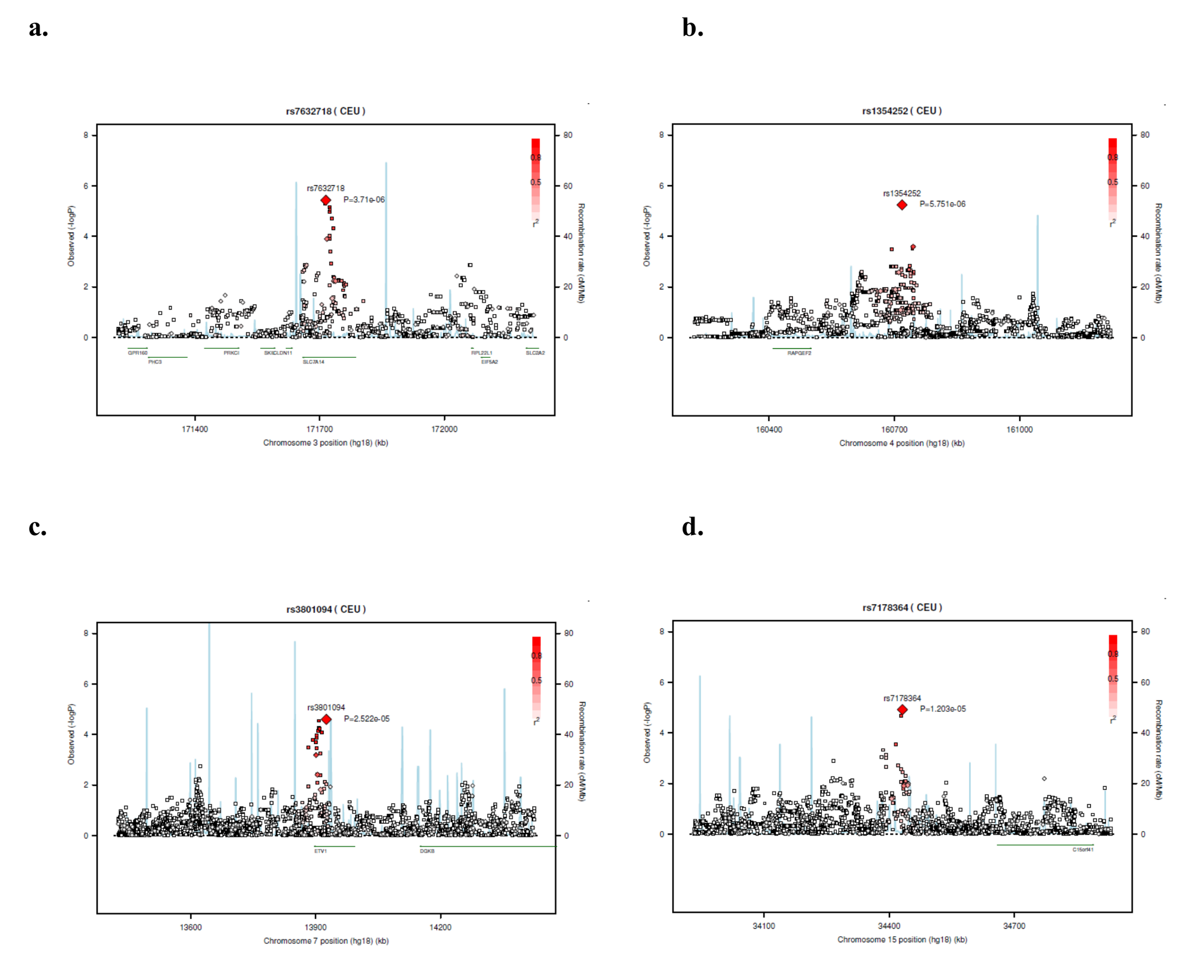

Supplement: Figure S3 — Regional association plots for additional 4 regions (a. 3q26.2, b. 4q32.1, c. 7p21.2, d. 15q14) replicating in the Australian study. Each SNP is plotted with respect to its chromosomal location (x axis) and its log10 transformed P value (y axis on the left) for associations with MPM. The tall blue spikes indicate the recombination rate (y axis on the right) at that region of the chromosome. The red-outlined diamond indicate the index SNP and other diamond indicate the genotyped SNPs, the squares indicate imputed SNPs using as reference 1000 Genomes Pilot 1 CEU population. LD values were calculated only on our control population (TIFF) [file pone.0061253.s003.tiff]

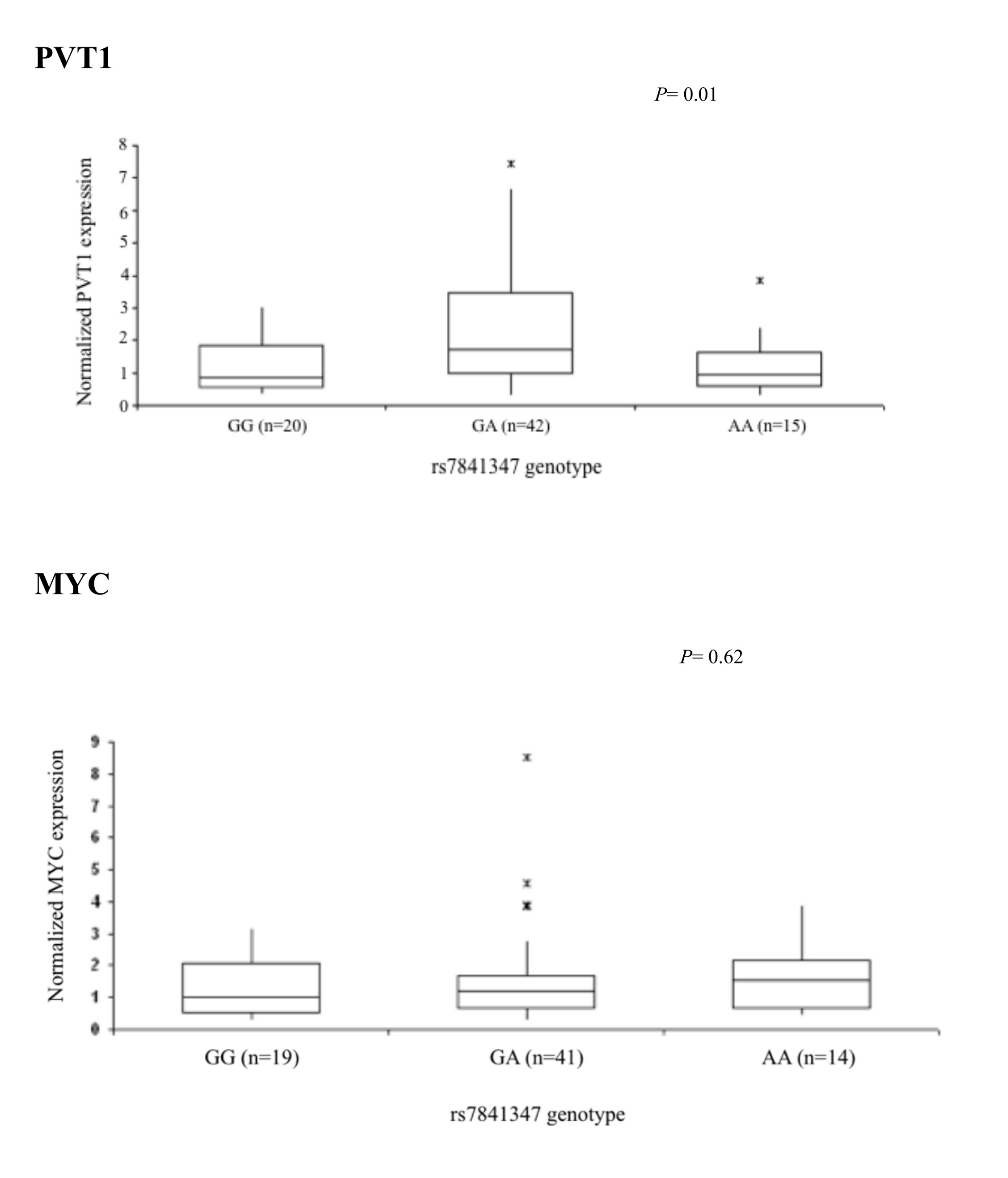

Supplement: Figure S4 — RT-PCR of PVT1 and MYC genes-expression levels in 79 normal pleural tissues expression levels across rs78941347 genotypes. (TIFF) [file pone.0061253.s004.tiff]
