# Supplementary material for: Genetic Variants Associated with Increased Risk of Malignant Pleural Mesothelioma: A Genome-Wide Association Study
Source: PLoS One. 2013 Apr 23;8(4):e61253. doi: 10.1371/journal.pone.0061253 (PMC3634031; doi:10.1371/journal.pone.0061253)
Supplement: Table S1 — Italian top 8 imputed SNP list. (DOCX) [file pone.0061253.s005.docx]

**Table S1 Italian top 8 imputed SNP list** (2-tailed logistic regression, n=759 overall, n=593 exposed only)

| **CHR Location** | **SNP** | **Ref. Allele** | **OR (95% CI)** | **P** | **Typed** | **Gene Name** | **Left Gene** | **Right Gene** | **Group** |
| --- | --- | --- | --- | --- | --- | --- | --- | --- | --- |
| 5q35.2 | rs6897549 | C | 0.53(0.41-0.69) | 1.48 x 10^-6^ | Imputed |  | *HMP19* | *MSX2* | OVERALL |
| 19q13.42 | rs71365421 | A | 0.35(0.23-0.54) | 2.17 x 10^-6^ | Imputed |  | *CACNG6* | *VSTM1* | OVERALL |
| 4q32.1 | rs1354252 | T | 2.14(1.54-2.97) | 5.75 x 10^-6^ | Imputed |  | *RAPGEF2* | *FSTL5* | EXPOSED |
| 14q11.2 | rs2236304 | G | 1.80(1.39-2.35) | 1.13 x 10^-5^ | Imputed | *MMP14* | *MRPL52* | *LRP10* | OVERALL |
| 15q14 | rs7178364 | A | 0.56(0.43-0.73) | 1.20 x 10^-5^ | Imputed |  | *MIR4510* | *C15orf41* | OVERALL |
| 12q23.3 | rs1072577 | A | 1.77(1.36-2.29) | 1.67 x 10^-5^ | Imputed |  | *CMKLR1* | *FICD* | EXPOSED |
| 9p24.1 | rs10815216 | C | 0.57(0.44-0.74) | 2.09 x 10^-5^ | Imputed | *PLGRKT/C9orf46* | *RLN1* | *CD274* | OVERALL |
| 7p22.2 | rs73034881 | A | 0.51(0.37-0.70) | 2.16 x 10^-5^ | Imputed |  | *SDK1* | *FOXK1* | OVERALL |
