# Supplementary material for: Genetic Variants Associated with Increased Risk of Malignant Pleural Mesothelioma: A Genome-Wide Association Study
Source: PLoS One. 2013 Apr 23;8(4):e61253. doi: 10.1371/journal.pone.0061253 (PMC3634031; doi:10.1371/journal.pone.0061253)
Supplement: Table S2 — Gene Set Enrichment Analysis. (DOCX) [file pone.0061253.s006.docx]

**Table S2 Gene Set Enrichment Analysis**

| **Gene Name** | **Entrez ID** | **Ensemble ID** | **Description** |
| --- | --- | --- | --- |
| *SHC4* | 399694 | ENSG00000185634 | SHC (Src homology 2 domain containing) family, member 4 |
| *MMP14* | 4323 | ENSG00000157227 | matrix metallopeptidase 14 (membrane-inserted) |
| *ETV1* | 2115 | ENSG00000006468 | ets variant 1 |
| *SLC7A14* | 57709 | ENSG00000013293 | solute carrier family 7 (cationic amino acid transporter, y+ system), member 14 |
| *ADAMTS2* | 9509 | ENSG00000087116 | ADAM metallopeptidase with thrombospondin type 1 motif, 2 |
| *PVT1* | 5820 | NA | Pvt1 oncogene (non-protein coding) |
| *THRB* | 7068 | ENSG00000151090 | thyroid hormone receptor, beta (erythroblastic leukemia viral (v-erb-a) oncogene homolog 2, avian) |
| *CEP350* | 9857 | ENSG00000135837 | centrosomal protein 350kDa |
| *C9orf46* | 55848 | ENSG00000107020 | chromosome 9 open reading frame 46 |
| **BIOLOGICAL PROCESSES** | | | |
| **lung development GO:0030324 rawP=5 x 10^-4^ adjP=8.7 x 10^-3^** | | | |
| *ADAMTS2* | | | |
| *MMP14* | | | |
| **respiratory tube development GO:0030323 rawP=5 x 10^-4^ adjP=8.7 x 10^-3^** | | | |
| *ADAMTS2* | | | |
| *MMP14* | | | |
| **respiratory system development GO:0060541 rawP=6 x 10^-3^ adjP=8.7 x 10^-3^** | | | |
| *ADAMTS2* | | | |
| *MMP14* | | | |
| **protein maturation by peptide bond cleavage GO:0051605 rawP=6 x 10^-4^ adjP=8.7 x 10^-3^** | | | |
| *ADAMTS2* | | | |
| *MMP14* | | | |
| **protein processing GO:0016485 rawP=8 x 10^-4^ adjP=9.3 x 10^-3^** | | | |
| *ADAMTS2* | | | |
| *MMP14* | | | |
| **protein maturation GO:0051604 rawP=8 x 10^-4^ adjP=9.7 x 10^-3^** | | | |
| *ADAMTS2* | | | |
| *MMP14* | | | |
| **tube development GO:0035295 rawP=2.27 x 10^-3^ adjP=2.24 x 10^-2^** | | | |
| *ADAMTS2* | | | |
| *MMP14* | | | |
| **molecular function metalloendopeptidase activity GO:0004222 rawP=7 x 10^-4^ adjP=1.4 x 10^-2^** | | | |
| *ADAMTS2* | | | |
| *MMP14* | | | |
| **molecular function metallopeptidase activity GO:0008237 rawP=2.1 x 10^-3^ adjP=2.1 x 10^-2^** | | | |
| *ADAMTS2* | | | |
| *MMP14* | | | |
