# Supplementary material for: Genetic Variants Associated with Increased Risk of Malignant Pleural Mesothelioma: A Genome-Wide Association Study
Source: PLoS One. 2013 Apr 23;8(4):e61253. doi: 10.1371/journal.pone.0061253 (PMC3634031; doi:10.1371/journal.pone.0061253)
Supplement: Table S3 — Significant Haplotype Results for 3p24 and 19q13.42 regions. (DOCX) [file pone.0061253.s007.docx]

**Table S3 Significant Haplotype Results for 3p24 and 19q13.42 regions.** N=759 subjects, 2 tailed logistic regression analysis.

| 3p24 |  |  |  |  |  |  |  |  |
| --- | --- | --- | --- | --- | --- | --- | --- | --- |
| **Num. SNPs** | **CHR** | **Position start** | **Position end** | **SNP start** | **SNP end** | **HAPLOTYPE** | **OR** | **P** |
| 5 | 3 | 24359009 | 24360079 | rs9833191 | rs1394770 | CGACA | 0.50 | 2.04 x 10^-7^ |
| 6 | 3 | 24358977 | 24360079 | chr3:24358977 | rs1394770 | CCGACA | 0.51 | 2.22 x 10^-7^ |
| 8 | 3 | 24358579 | 24360079 | rs13085323 | rs1394770 | AGCCGACA | 0.51 | 2.23 x 10^-7^ |
| 7 | 3 | 24358645 | 24360079 | rs13085964 | rs1394770 | GCCGACA | 0.51 | 2.30 x 10^-7^ |
| 3 | 3 | 24359009 | 24359358 | rs9833191 | rs9875048 | CGA | 0.51 | 2.66 x 10^-7^ |
| 4 | 3 | 24359009 | 24359964 | rs9833191 | chr3:24359964 | CGAC | 0.51 | 2.66 x 10^-7^ |
| 6 | 3 | 24358579 | 24359358 | rs13085323 | rs9875048 | AGCCGA | 0.51 | 2.73 x 10^-7^ |
| 7 | 3 | 24358579 | 24359964 | rs13085323 | chr3:24359964 | AGCCGAC | 0.51 | 2.73 x 10^-7^ |
| 4 | 3 | 24358977 | 24359358 | chr3:24358977 | rs9875048 | CCGA | 0.51 | 2.75 x 10^-7^ |
| 5 | 3 | 24358977 | 24359964 | chr3:24358977 | chr3:24359964 | CCGAC | 0.51 | 2.75 x 10^-7^ |
| 5 | 3 | 24358645 | 24359358 | rs13085964 | rs9875048 | GCCGA | 0.51 | 2.76 x 10^-7^ |
| 6 | 3 | 24358645 | 24359964 | rs13085964 | chr3:24359964 | GCCGAC | 0.51 | 2.76 x 10^-7^ |
| 9 | 3 | 24358351 | 24360079 | rs13318276 | rs1394770 | CAGCCGACA | 0.51 | 3.53 x 10^-7^ |
| 2 | 3 | 24358977 | 24359009 | chr3:24358977 | rs9833191 | CC | 0.52 | 4.50 x 10^-7^ |
| 2 | 3 | 24359009 | 24359334 | rs9833191 | chr3:24359334 | CG | 0.52 | 4.50 x 10^-7^ |
| 3 | 3 | 24358645 | 24359009 | rs13085964 | rs9833191 | GCC | 0.52 | 4.50 x 10^-7^ |
| 3 | 3 | 24358977 | 24359334 | chr3:24358977 | chr3:24359334 | CCG | 0.52 | 4.50 x 10^-7^ |
| 4 | 3 | 24358645 | 24359334 | rs13085964 | chr3:24359334 | GCCG | 0.52 | 4.50 x 10^-7^ |
| 7 | 3 | 24358351 | 24359358 | rs13318276 | rs9875048 | CAGCCGA | 0.52 | 4.69 x 10^-7^ |
| 8 | 3 | 24358351 | 24359964 | rs13318276 | chr3:24359964 | CAGCCGAC | 0.52 | 4.69 x 10^-7^ |
| 5 | 3 | 24358579 | 24359334 | rs13085323 | chr3:24359334 | AGCCG | 0.53 | 8.23 x 10^-7^ |
| 4 | 3 | 24358579 | 24359009 | rs13085323 | rs9833191 | AGCC | 0.53 | 8.64 x 10^-7^ |
| 19q13.42 |  |  |  |  |  |  |  |  |
| **Num. SNPs** | **CHR** | **Position start** | **Position end** | **SNP start** | **SNP end** | **HAPLOTYPE** | **OR** | **P** |
| 5 | 19 | 59228967 | 59232479 | rs4239595 | chr19:59232479 | ATGTA | 0.33 | 7.02 x 10^-7^ |
| 6 | 19 | 59228798 | 59232479 | rs2448290 | chr19:59232479 | CATGTA | 0.33 | 7.02 x 10^-7^ |
| 6 | 19 | 59228967 | 59232591 | rs4239595 | rs287128 | ATGTAG | 0.33 | 7.02 x 10^-7^ |
| 7 | 19 | 59228798 | 59232591 | rs2448290 | rs287128 | CATGTAG | 0.33 | 7.02 x 10^-7^ |
| 4 | 19 | 59229149 | 59232479 | rs8101204 | chr19:59232479 | TGTA | 0.33 | 7.06 x 10^-7^ |
| 5 | 19 | 59229149 | 59232591 | rs8101204 | rs287128 | TGTAG | 0.33 | 7.06 x 10^-7^ |
| 7 | 19 | 59227385 | 59232479 | rs35474370 | chr19:59232479 | CCATGTA | 0.33 | 8.35 x 10^-7^ |
| 8 | 19 | 59226644 | 59232479 | rs583273 | chr19:59232479 | GCCATGTA | 0.33 | 8.35 x 10^-7^ |
| 9 | 19 | 59226281 | 59232479 | rs584734 | chr19:59232479 | AGCCATGTA | 0.33 | 8.35 x 10^-7^ |
| 8 | 19 | 59227385 | 59232591 | rs35474370 | rs287128 | CCATGTAG | 0.33 | 9.06 x 10^-7^ |
| 9 | 19 | 59226644 | 59232591 | rs583273 | rs287128 | GCCATGTAG | 0.33 | 9.06 x 10^-7^ |
| 10 | 19 | 59226281 | 59232591 | rs584734 | rs287128 | AGCCATGTAG | 0.33 | 9.06 x 10^-7^ |
| 2 | 19 | 59230439 | 59232479 | rs12610534 | chr19:59232479 | TA | 0.34 | 9.73 x 10^-7^ |
| 2 | 19 | 59232479 | 59232591 | chr19:59232479 | rs287128 | AG | 0.34 | 9.73 x 10^-7^ |
| 3 | 19 | 59229819 | 59232479 | rs2438043 | chr19:59232479 | GTA | 0.34 | 9.73 x 10^-7^ |
| 3 | 19 | 59230439 | 59232591 | rs12610534 | rs287128 | TAG | 0.34 | 9.73 x 10^-7^ |
| 4 | 19 | 59229819 | 59232591 | rs2438043 | rs287128 | GTAG | 0.34 | 9.73 x 10^-7^ |
