# Supplementary material for: Genetic Variants Associated with Increased Risk of Malignant Pleural Mesothelioma: A Genome-Wide Association Study
Source: PLoS One. 2013 Apr 23;8(4):e61253. doi: 10.1371/journal.pone.0061253 (PMC3634031; doi:10.1371/journal.pone.0061253)
Supplement: Table S4 — Replication of the 12 genotyped Italian top SNPs on GUARD-BHS Study. (DOCX) [file pone.0061253.s008.docx]

**Table S4 Replication of the 12 genotyped Italian top SNPs on GUARD-BHS Study** (2-tailed logistic regression analysis)

| **CHR Location** | **SNP** | **Ref. Allele** | **OR (95% CI)** | **P** |
| --- | --- | --- | --- | --- |
| 6q21 | rs742109 | A | 0.99(0.83-1.19) | 0.95 |
| 5q23.1 | rs1508805 | T | 1.13(0.92-1.40) | 0.24 |
| 1q25.2 | rs2501618 | A | 0.99(0.77-1.26) | 0.93 |
| 4q22.1 | rs4290865 | A | 0.79(0.62-1.00) | 0.05 |
| 13q14.3 | rs9536579 | A | 1.03(0.82-1.28) | 0.81 |
| 7p21.2 | rs3801094 | A | 0.94(0.88-1.00) | 0.53 |
| 8q24.21 | rs7841347 | A | 0.92(0.77-1.10) | 0.37 |
| 15q21.1 | rs10519201 | C | 0.95(0.72-1.26) | 0.74 |
| 22q12.3 | rs5756444 | G | 1.03(0.86-1.24) | 0.75 |
| 3p24.2 | rs9833191 | C | 1.03(0.85-1.23) | 0.77 |
| 5q35.2 | rs6897549 | C | 0.87(0.71-1.06) | 0.18 |
| 3q26.2 | rs7632718 | C | 0.99(0.83-1.18) | 0.91 |
