# Supplementary material for: Genetic Variants Associated with Increased Risk of Malignant Pleural Mesothelioma: A Genome-Wide Association Study
Source: PLoS One. 2013 Apr 23;8(4):e61253. doi: 10.1371/journal.pone.0061253 (PMC3634031; doi:10.1371/journal.pone.0061253)
Supplement: Table S5 — Meta-analysis of Italian and Australian studies for the top 12 genotyped Italian SNPs. (DOCX) [file pone.0061253.s009.docx]

**Table S5 Meta-analysis of Italian and Australian studies for the top 12 genotyped Italian SNPs**

| **SNP** | **Ref. Allele** | **OR (95% CI)** | **P** | **I^2^** | **Num. Studies** |
| --- | --- | --- | --- | --- | --- |
| rs4290865 | A | 1.24 (0.51-3.05) | 0.63 | 0.95 | 2 |
| rs9536579 | A | 0.74 (0.40-1.41) | 0.37 | 0.92 | 2 |
| rs10519201 | G | 0.72(0.41-1.26) | 0.24 | 0.88 | 2 |
| rs1508805 | A | 1.44(0.89-2.33) | 0.14 | 0.87 | 2 |
| rs2501618 | A | 1.45(0.67-3.15) | 0.34 | 0.92 | 2 |
| rs3801094 | A | 1.27(0.69-2.34) | 0.43 | 0.93 | 2 |
| rs4701085 | A | 0.77(0.39-1.51) | 0.44 | 0.94 | 2 |
| rs5756444 | G | 0.79(0.46-1.35) | 0.39 | 0.92 | 2 |
| rs742109 | C | 1.33(0.74-2.41) | 0.34 | 0.93 | 2 |
| rs7632718 | G | 0.71(0.37-1.35) | 0.30 | 0.97 | 2 |
| rs7841347 | A | 0.75(0.49-1.14) | 0.17 | 0.87 | 2 |
| rs9833191 | T | 1.24(0.74-2.09) | 0.41 | 0.62 | 2 |
