# Supplementary material for: Genetic Variants Associated with Increased Risk of Malignant Pleural Mesothelioma: A Genome-Wide Association Study
Source: PLoS One. 2013 Apr 23;8(4):e61253. doi: 10.1371/journal.pone.0061253 (PMC3634031; doi:10.1371/journal.pone.0061253)
Supplement: Text S1 — Supplementary Materials. (DOCX) [file pone.0061253.s010.docx]

**SUPPLEMENTARY MATERIALS**

**Study samples description**

*Casale Monferrato panel*

The Casale Monferrato MPM study is a population-based case-control study. The detailed study design was previously reported[[1](#_ENREF_1)].

In brief, the cases group included subjects with histologically diagnosed MPM resident in the Local Health Authority (LHA) of Casale Monferrato. This area was characterized by widespread environmental exposure of the general population to asbestos fibers due to the presence in that area of an asbestos cement factory active from 1907 to 1986[[2](#_ENREF_2)].

Two controls per patient, matched for age (±18 months) and gender, were randomly selected from the local population using the Casale Monferrato LHA rosters.

Cases and controls were recruited between January 2001 and December 2010 for a total of 334 eligible cases and 552 controls.

Trained personnel submitted a standard questionnaire to MPM cases and controls to collect information about demographics, life-style, occupational history and asbestos exposure[[2](#_ENREF_2)]. The evaluation of asbestos exposure was performed blindly by an industrial hygienist (D.M.) and summarized as: “certain occupational”, “probable occupational”, “possible occupational”, “household exposure”, “environmental exposure” and “no evidence of exposure”. Exposure was further considered as a categorical variable (high, intermediate/low exposed and non-exposed). All subjects signed an informed consent form before the interview. Ninety-three cases and 300 controls refused to provide a blood sample. Consequently, the study included a total of 241 MPM patients and 252 population controls whose main characteristics are summarized in Table 1. Most cases and controls had Italian nationality and were of Caucasian ethnicity.

Blood samples were collected before therapy in vacutainers with ethylenediaminetetraacetic acid (EDTA) and stored at -20°C until use.

*Turin panel*

The Turin MPM study is an hospital-based case-control study. The detailed study design was reported previously[[3](#_ENREF_3)].

The cases group included subjects with a histologically confirmed diagnosis of MPM admitted to the chest surgery units of the San Giovanni Battista (Turin) or the San Luigi Gonzaga (Orbassano) hospitals between January 2004 and December 2006. All cases were resident in Turin or in the province of Turin at the time of diagnosis. After a new case identification, a matched control was chosen among general medicine or urology patients within the same hospital, living in Turin or in the province of Turin at the time of admission, and not affected by neoplastic or respiratory conditions. The original study design envisaged the selection of one control per patient matched by age (± 24 months) and gender, but no eligible control was found for 35 cases. Thus, the Turin panel included 91 MPM patients and 56 controls. Patients and controls were interviewed using the same questionnaire described above. Asbestos exposure was evaluated as for the Casale Monferrato group by the same industrial hygienist (D.M.). All subjects signed an informed consent form and provided a blood sample that was collected and stored as reported above. All participants had Italian nationality and were of Caucasian ethnicity (Table 1).

*Liguria panel*

The cases group included incident MPM cases consecutively recruited between 1999 and 2006 from pneumology departments of three general hospitals located in Liguria region (Genoa, La Spezia). The MPM patients derived from the Cancer of RESpiratory Tract (CREST) biobank, established in 1996 within the National Cancer Research Institute of Genoa. The detailed study design was reported previously[[4](#_ENREF_4)]. In this area, MPM incidence is particularly high because of the dense concentration of asbestos-related industries, including an extensive shipyard activity[[5](#_ENREF_5)]. Controls are healthy subjects (blood donors or volunteers from recreational associations) or patients hospitalized for non-neoplastic and non-respiratory conditions (mostly traumatic diseases or eye diseases).

All subjects in the study signed an informed consent form. An extensive questionnaire was administered by trained personnel, and detailed information was collected including socio-demographic data, residence history, current and former occupations or hobbies in which the subject may have been in contact with asbestos, lifestyle information (tobacco smoke and diet), medical history and family history of cancer in first degree relatives. Blood samples were collected and stored as reported above for Casale and Turin panels. The Genoa panel included 75 MPM patients and 81 controls. Controls were matched for age (±24months) and gender.

**DNA extraction and preparation**

For the Italian sample, genomic DNA was extracted from 200 µl peripheral whole blood by an on-column DNA purification method (QIAamp DNA Blood Mini Kit, QIAGEN GmbH, Germany) retrieving the amount of DNA within the range of 4-12 µg.

DNA integrity was checked by standard electrophoresis on a 1% agarose gel in TBE 0.5X buffer.

DNA purity and concentration were assessed by a NanoDrop 8000 Spectrophotometer (Thermo Fisher Scientific Inc.).

Two hundred nanograms of genomic DNA for each sample were used for a whole-genome genotyping assay on an Illumina genotyping BeadChip: 716 samples were tested on a HumanCNV370-Quad, while further 80 samples were tested on a Human610-Quad (which includes 100% of the HumanCNV370 BeadChip SNPs) due to discontinuation of the HumanCNV370-Quad from Illumina Inc. (San Diego, CA).

Individual genotypes were assessed by the dedicated GenomeStudio V2011.1 software with Genotyping Module v1.9.4 (Illumina Inc., San Diego, CA).

**Gene Expression Analysis**

*Gene expression analysis in pleural tissues*

Seventy-nine samples of normal pleura were obtained from donors that underwent thoracoscopy for conditions different from MPM, and signed an informed consent.

DNA was extracted from frozen pleural tissues by QIAamp® DNA Mini Kit (QIAGEN) according to the manufacturer’s protocol and genotyped for rs9833191 (*THRB*) and rs7841347 (*PVT1*) by Sanger sequencing.

Total RNA was extracted by RNeasy® Plus Mini Kit (QIAGEN) according to the manufacturer’s protocol and cDNA was retrotranscribed from 150ng of total RNA using random primers (High Capacity cDNA Reverse Transcription Kit, Applied Biosystems).

The mRNA levels of *THRB*, *PVT1* and *MYC* were measured by quantitative real-time PCR (qPCR).

By evaluation of thirty-two reference genes with the geNorm software, we selected *UBC* and *RPL37A* as the most stable genes in normal pleura. *THRB*, *PVT1* and *MYC* mRNA levels were normalized against *UBC* as reference gene, whereas *MYC* mRNA levels were normalized using both *UBC* and *RPL37A*.

Relative gene expression quantification for target genes and reference genes was carried out in triplicate, with TaqMan® chemistry using ABI Prism 7000 Sequence Detection System (Applied Biosystems).

To assess variation between experiments, a standard cDNA was included in each plate.

The analyses were carried out using the comparative C_t_ method (∆∆C_t_ method) (User Bulletin #2: ABI PRISM 7700 Sequence Detection System). The threshold cycle (C_t_), is defined as the number of cycles required for the fluorescent signal to cross a threshold (i.e. exceeds background level). C_t_ levels are inversely proportional to the amount of mRNA in the sample (i.e. the lower C_t_ level the greater the amount of mRNA in the sample).

C_t_ level was determined for each gene and the relative expression was then estimated by calculating the dC_t_ value, defined as the difference in the C_t_ value for the target gene (*THRB*, *PVT*1 or *MYC*) and the reference genes (*UBC* and *RPL37A*).

Differences in the distribution of expression by SNP genotype were compared using the ANOVA test.

Melaiu and colleagues[[6](#_ENREF_6)] produced an up to date review of MPM studies involving microarray techniques. This summary work helped to identify deregulated genes in pleural mesothelioma. In their supplementary materials we found that three of our candidate gene transcripts (*MMP14, THRB* and *MYC*) were reported in at least four MPM studies and were significantly different (normal *vs* affected samples) in at least one. Thus, we verified the expression levels of our geneset in the following datasets: i) GSE12345[[7](#_ENREF_7)] made up of 13 samples (4 normal pleural tissues and 9 MPM) ii) GSE2549[[8](#_ENREF_8)] where we included MPM tumor specimens (n=40), normal lung specimens (n=4) and normal pleura specimens (n=5) iii) the GSK Cancer Cell Line Genomic Profiling Data[[9](#_ENREF_9)] iv) MTAB47 data from genome-wide profile of pleural mesothelioma versus parietal and visceral pleura[[10](#_ENREF_10)].

In MTAB47 data, three out of four probes in *CEP350* gene showed high expression levels both in normal tissue (log2 median intensity value: 8.2, 9.85, 9.51 respectively) and in mesothelioma (log2 median intensity value: 8.28, 9.91, 9.42 respectively). Similar behavior, but at a lower level of expression, can be observed in GSE2549 (log2 median intensity value: 7, 7.3 in MPM and normal tissue respectively). For *ADAMTS2* gene there was evidence (median intensity value: 98.3, 96.7, 124.8, 39 in mesothelioma cell line versus 241.2, 229.3, 189.2, 828.9 in normal tissue) of under expression in mesothelioma versus normal tissue in GSK data. This result could not be confirmed in other data where the T-statistic *P* for differential expression were ranging from 0.208 to 0.796. *ETV1* in GSE2549 showed low levels of expression for all its probes (median intensity value does not exceed 110 for both MPM and normal), with a slight tendency to be under-expressed in malignant tissue (three probes reached significant *P*, in the range 0.002 – 0.038 for the T-statistic). *PVT1* in GSE2549 showed very low levels of expression both in MPM and in normal tissue (median intensity value: 17.4, 66.4 respectively in MPM and normal, the T-statistic is still significant at 0.0004 level). For *SHC4* gene there were no data in pleural tissue, whereas its paralog *SHC1* is significantly up-regulated (T-statistic *P* = 0.013) in MPM *vs* normal tissue (GSE2549). *THRB* gene expression was significantly different in normal pleural tissue *vs* MPM (GSE12345: log fold change = 4.8, T-statistic = -5.76, *P* = 1 x 10^-4^), but expression levels were quite low (median intensity value of 5.6 in normal pleura 2.9 in mesothelioma). For *SLC7A14* gene there were no data in pleural tissue. *MMP14* showed a significant difference of expression in normal pleural tissue *vs* MPM (GSE12345) (log fold change = -3.9, T-Statistic = 7.51, *P* = 9 x 10^-6^). No data were reported for *C9ORF46* in pleural tissue.

**References**

1. Dianzani I, Gibello L, Biava A, Giordano M, Bertolotti M, et al. (2006) Polymorphisms in DNA repair genes as risk factors for asbestos-related malignant mesothelioma in a general population study. Mutation Research - Fundamental and Molecular Mechanisms of Mutagenesis 599: 124-134.

2. Magnani C, Dalmasso P, Biggeri A, Ivaldi C, Mirabelli D, et al. (2001) Increased risk of malignant mesothelioma of the pleura after residential or domestic exposure to asbestos: A case-control study in Casale Monferrato, Italy. Environmental Health Perspectives 109: 915-919.

3. Betti M, Ferrante D, Padoan M, Guarrera S, Giordano M, et al. (2011) XRCC1 and ERCC1 variants modify malignant mesothelioma risk: A case-control study. Mutation Research - Fundamental and Molecular Mechanisms of Mutagenesis 708: 11-20.

4. Ugolini D, Neri M, Canessa PA, Casilli C, Catrambone G, et al. (2008) The CREST biorepository: a tool for molecular epidemiology and translational studies on malignant mesothelioma, lung cancer, and other respiratory tract diseases. Cancer epidemiology, biomarkers & prevention : a publication of the American Association for Cancer Research, cosponsored by the American Society of Preventive Oncology 17: 3013-3019.

5. Gennaro V, Ugolini D, Viarengo P, Benfatto L, Bianchelli M, et al. (2005) Incidence of pleural mesothelioma in Liguria Region, Italy (1996-2002). Eur J Cancer 41: 2709-2714.

6. Melaiu O, Cristaudo A, Melissari E, Di Russo M, Bonotti A, et al. (2011) A review of transcriptome studies combined with data mining reveals novel potential markers of malignant pleural mesothelioma. Mutation research.

7. Crispi S, Calogero RA, Santini M, Mellone P, Vincenzi B, et al. (2009) Global gene expression profiling of human pleural mesotheliomas: identification of matrix metalloproteinase 14 (MMP-14) as potential tumour target. PLoS One 4: e7016.

8. Gordon GJ, Rockwell GN, Jensen RV, Rheinwald JG, Glickman JN, et al. (2005) Identification of novel candidate oncogenes and tumor suppressors in malignant pleural mesothelioma using large-scale transcriptional profiling. Am J Pathol 166: 1827-1840.

9. Greshock J, Bachman KE, Degenhardt YY, Jing J, Wen YH, et al. (2010) Molecular target class is predictive of in vitro response profile. Cancer Res 70: 3677-3686.

10. Roe OD, Anderssen E, Helge E, Pettersen CH, Olsen KS, et al. (2009) Genome-wide profile of pleural mesothelioma versus parietal and visceral pleura: the emerging gene portrait of the mesothelioma phenotype. PloS one 4: e6554.
