# Supplementary material for: Genetic Variants Associated with Increased Risk of Malignant Pleural Mesothelioma: A Genome-Wide Association Study
Source: PLoS One. 2013 Apr 23;8(4):e61253. doi: 10.1371/journal.pone.0061253 (PMC3634031; doi:10.1371/journal.pone.0061253)
Supplement: File S1 — URLs. (DOCX) [file pone.0061253.s011.docx]

**File S1. URLs**

GO, http://www.geneontology.org; Impute, http://mathgen.stats.ox.ac.uk/impute/impute_v2.html; GSEA, <http://www.broadinstitute.org/gsea>;

GWAMA, http://www.well.ox.ac.uk/gwama; 1KGenome, <http://www.1000genomes.org>;

HapMap, http://www.hapmap.org; R, http://cran.r-project.org;

KEGG, http://www.genome.jp/kegg; REACTOME, <http://www.reactome.org>;

PLINK; <http://pngu.mgh.harward.edu/~purcell/plink>.
